# Free-Field Reciprocity Calibration Of Microphones

**DOI:** 10.6028/jres.092.013

**Published:** 1987-04-01

**Authors:** Edwin D. Burnett, Victor Nedzelnitsky

**Affiliations:** National Bureau of Standards Gaithersburg, MD 20899

**Keywords:** acoustic calibrations, acoustic measurements, anechoic chamber measurements, calibration, free-field microphone calibration, metrology, microphone calibration, plane-wave free-field acoustic measurements, reciprocity calibration methods, standards: acoustical, traceability: acoustical measurements, transducer calibration (reciprocity)

## Abstract

Standardized methods for the primary free-field calibration of laboratory standard microphones deal with Type L (ANSI S1.10-1967, R1977) “one-inch” diameter microphones. However, the use of “1/2-inch” diameter microphones for measurement of the sound pressure level in acoustic fields is increasing. Consequently, the NBS has developed a fixed-cost measurement service for the free-field calibration of these microphones by the reciprocity method over the range 2.5 kHz to 20 kHz. For this service, the apparatus and procedures, including essential properties of the anechoic chamber in which the calibrations are performed, are described. Opportunities for improvements are noted. The frequency-dependent positions of the apparent acoustic centers of the microphones were obtained. The overall uncertainty estimate for free-field calibration, expressed as the sum of the magnitude of credible bounds on the systematic component (s) and the random component (2*σ*, where *σ* is the standard deviation) is 0.16 dB or better (s=0.06 dB, 2*σ*=0.10 dB) at frequencies 1.25 kHz ⩽*f*⩽ 5kHz, and 0.07 dB or better (s=0.02 dB, 2*σ*=0.05 dB) for 5 kHz ⩽*f*⩽ 20 kHz. Comparison for given microphones of the measured difference between free-field and pressure response levels with the difference calculated by diffraction theory (derived by Matsui) indicates agreement of 0.16 dB or better in the low-frequency range (1.25 kHz to about 4 kHz) where free-field reciprocity measurements encounter the greatest experimental difficulties. This agreement is consistent with the estimated uncertainties of free-field and pressure calibration by the reciprocity method.

## 1. Introduction

The two different electroacoustical responses (sensitivities) that are most frequently requested of the microphone calibration services at the National Bureau of Standards (NBS) are the pressure response (pressure sensitivity) and the free field response (free-field sensitivity). Both are usually expressed in terms of the ratio of the open-circuit voltage (at the output of the microphone) to a sound pressure in the acoustic field in which the microphone is located, for a given frequency of excitation in the sinusoidal steady state (units: V/Pa).

Throughout this paper, the voltages, currents and sound pressures are the rms (root-mean-square) values of the sinusoidal quantities. Although these quantities will in general have phase differences between them, such differences do not figure in standardized calibration by the reciprocity method. Calibration for phase response by this method is a subject for further research and standards yet to be determined.

At a given frequency in the sinusoidal steady state, the pressure response is the ratio of the output voltage of the microphone to the sound pressure uniformly distributed over the exterior surface of the microphone diaphragm. The pressure response is generally determined using the reciprocity technique and acoustic couplers essentially enclosing a coupling cavity into which the microphone diaphragms are introduced. The pressure response is needed for measurement of the sound pressure level in cavities or couplers, as in the calibration of audiometric and other earphones and many types of acoustic calibrators.

At a given frequency in the sinusoidal steady state, the free-field response is the ratio of the output voltage of the microphone to the sound pressure which existed at the microphone’s acoustic center (or specified reference point) prior to the introduction of the microphone into the path of a plane progressive sound wave. The direction of propagation of this wave has a specified orientation with respect to the principal axis of symmetry of the microphone, and for the most precise laboratory standards purposes is usually chosen parallel to this axis, so that the direction of propagation is perpendicular to and toward the diaphragm surface of the microphone. This orientation is usually termed normal incidence. The free-field response differs from the pressure response due to diffraction effects associated with the relation between the wavelength of sound and the physical dimensions of the microphone and its mounting, with the difference becoming large at frequencies sufficiently high that a wavelength becomes comparable to or smaller than these dimensions. For practical laboratory standard microphones, these diffraction effects are sufficiently large at frequencies of interest that free-field calibrations are needed for the most accurate acoustical measurements under free-field conditions.

Currently applicable standardized laboratory methods for the primary free-field calibration of 1-inch nominal diameter laboratory standard microphones (hereafter termed “1-inch” microphones; their actual diameter is about 0.936 inch or 23.77 mm) by the reciprocity method have been available for over a decade. However, many of the microphones used for precision acoustical measurements have a nominal diameter of one-half inch (hereafter termed 1/2-inch microphones; the actual diameter at the mounting base is about 0.500 inch or 12.7 mm). Such microphones are usually used for determining the sound pressure level (SPL) in acoustic fields, rather than in cavities or couplers. The use of the smaller size microphones can be expected to increase with the availability of 1/2-inch microphones which are as sensitive as those of the “1-inch” size. Thus, the capacity to perform free-field reciprocity calibrations for 1/2-inch microphones has been added to the NBS fixed-cost microphone calibration services. Refinements in electronic instrumentation, apparatus, and procedure have enabled these services to attain accuracies comparable to or better than those that had been achieved at NBS for calibration of “1-inch” microphones. The purpose of this paper is to describe the methods, apparatus, procedures, and uncertainties associated with the free-field reciprocity calibration of 1/2-inch microphones at NBS.

## 2. Method

The theory for reciprocity calibrations using harmonic excitation has been well developed [1–5]^1^ and appears in both a domestic (ANSI) standard [3] and an international (IEC) standard [5]. This theory will not be repeated in depth here. An extensive bibliography is found in reference [3]. Briefly, three microphones are used for a calibration. The procedure of specific measurements performed on the three microphones can be chosen either to determine the sensitivities of all three microphones, in which case we denote the procedure as the “three-microphone method,” or to determine the sensitivities of two of the three microphones with the third microphone used only as a source, in which case we denote the procedure as the “two-microphone method.”

For the three-microphone method, the microphones are used sequentially as source and receiver. At each frequency of interest for each of the three sequentially performed measurements, the ratio of the receiver output voltage to the source input current is determined. These results are combined with reciprocity theory and the values of other pertinent parameters of the method to determine the free-field sensitivities of the three microphones.

For the two-microphone method, the ratio of the receiving output voltage from each of the two microphones being calibrated to the voltage applied to the third microphone driven to act as a sound source is measured sequentially; first, for one receiving microphone at all frequencies of calibration, next, for the other receiving microphone, after it has been substituted for the first receiving microphone. These ratios are then combined to determine, at each frequency of interest for a given free-field sound pressure, the ratio of the output voltages of the two microphones being calibrated. This ratio effectively determines the ratio of the sensitivities of these two microphones. One of these microphones is then used as the receiver and the other as the source, and the ratio of the receiver voltage to source drive current is determined. This ratio is combined with the ratio of sensitivities, reciprocity theory, and the values of other pertinent parameters of the method to determine the sensitivities of the two microphones being calibrated.

The techniques used by the NBS will be introduced by first considering the formulas from which the microphone sensitivities are determined from the measurements for the three-microphone method and the two-microphone method given in reference [5]. Using the notation of reference [3], when applicable, the three-microphone method yields the following equation for the magnitude of the sensitivity of the microphone (a):

$$\begin{array}{l} |M_{a}|=\{\frac{2}{\rho_{o}f}\frac{r_{\text{ab}}r_{\text{da}}}{r_{\text{bd}}}\frac{\left|e_{b}/i_{a}|\cdot |e_{a}/i_{d}\right|}{\left|e_{d}/i_{b}\right|}\\ \exp[\alpha (r_{\text{ab}}+r_{\text{da}}-r_{\text{bd}})]{\}}^{1/2} \end{array}$$ (1)

where *r_xy_*(*x,y* =a,b,d) is the distance between the acoustic centers (as defined in reference [3]) of microphone *x* and microphone *y*,*f* is the driving frequency, *i_y_* is the complex amplitude of the driving current through microphone *y, e_x_* is the complex amplitude of the output voltage of microphone *x, a* is the attenuation coefficient of sound in air at frequency *f*, and *ρ*_0_ is the ambient density of air. For the two-microphone method the magnitude of the sensitivity of microphone (a) is given by

$$|M_{\text{fa}}|={\left[\frac{2}{\rho_{o}f}r_{\text{ab}}|e_{b}/i_{a}|\cdot |e_{a}/e_{b}|\exp\;(\alpha r_{\text{ab}})\right]}^{1/2}$$ (2)

We first consider the merits of each of these methods. If three microphones are being calibrated at the same time, the three-microphone method gives directly the sensitivities for all three microphones. However, the two-microphone method gives a result which, for a given expenditure of labor and cost of apparatus, is more accurate in many practical cases. With this method only one measurement of current, which involves measurement of microphone driving-point electrical impedance (sometimes expressed in terms of the microphone capacitance) in our technique [4], is required instead of three. The uncertainty due to error in this particular portion of the measurement is reduced. Significant advantage is found when measuring the output of two conventional, low-sensitivity (i.e., with response levels of about −38 dB re 1V/Pa, as distinguished from high-sensitivity, with response levels about −26 dB re 1V/Pa) 1/2-inch microphones. The magnitude ratio of the output voltages of the two microphones [|*e*_a_/*e*_b_| in eq (2)] can be determined using a high-sensitivity 1/2-inch microphone or a “1-inch” microphone as the source. With either of these microphone types, the SPL will be more than 10 dB higher over most of the frequency range than it otherwise would be for two of the three required output level determinations. This higher SPL produces a higher signal-to-noise ratio which gives a better calibration accuracy at the lower frequencies of calibration (for which the signal-to-noise ratio is lower) and reduces the time required for making the measurement by reducing the signal processing time required to reduce uncertainty introduced by the noise. The two-microphone method will be discussed throughout the remainder of this report.

Accurate methods and apparatus for deriving the calibration, using attenuator settings to determine the ratios of the source voltages to the receiver voltages and the driving-point electrical impedance of the source (reversible) microphone (which is derived from an attenuator setting and a known resistance) have been described [3,4]. Specific adaptation of these methods to free-field measurements and a description of the apparatus employed at the NBS are provided in section 3 of this paper.

Using eq (2) and its counterpart for |*M*_fb_| [3,5], the equation for the response level *R*_fx_ (*x*=a,b) in dB re 1V/Pa can be expressed as

$$\begin{array}{l} R_{\text{fa}}=20\;\log_{10}[M_{\text{fa}}/\left(1V/\text{Pa}\right)]=\frac{1}{2}[A_{\text{bd}}-A_{\text{ad}}-A_{\text{ba}}]\\ \;+D_{o}\;\text{db re}\;1V/\text{Pa} \end{array}$$ (3)

$$\begin{array}{l} R_{\text{fb}}=20\;\log_{10}[M_{\text{fb}}/\left(1V/\text{Pa}\right)]=\frac{1}{2}[A_{\text{ad}}-A_{\text{bd}}-A_{\text{ba}}]\\ \;+D_{o}\;\text{db re}\;1V/\text{Pa} \end{array}$$ (4)

where

$$D_{o}=10{\;\log}_{10}\left[\frac{r_{\text{ab}}T}{p_{s}C_{a}f^{2}}\right]+4.343\;\alpha \;r_{\text{ab}}+19.605$$ (5)

*r*_ab_ is the distance in meters between the acoustic centers of microphones (a) and (b)
*p_s_* is the barometric pressure in pascals
*T* is the absolute temperature in kelvins

$$C_{a}=\frac{1}{2\pi f\left|Z_{a}\right|.}$$ (6)

where |Z_a_| is the magnitude of the driving-point electrical impedance of the reciprocal microphone (used as a sound source) in ohms.

The quantities *A_xy_* (*x* =a,b, *y* =a,d), which are attenuator settings of the calibration apparatus in decibels, are defined in section 3.1.

The accuracy of the calibration can be improved by reversing the functions of the microphones (a) and (b), taking the second set of measurements, and averaging the results of these two sets.

## 3. Apparatus

### 3.1 Block Diagram

**Figure 1:**
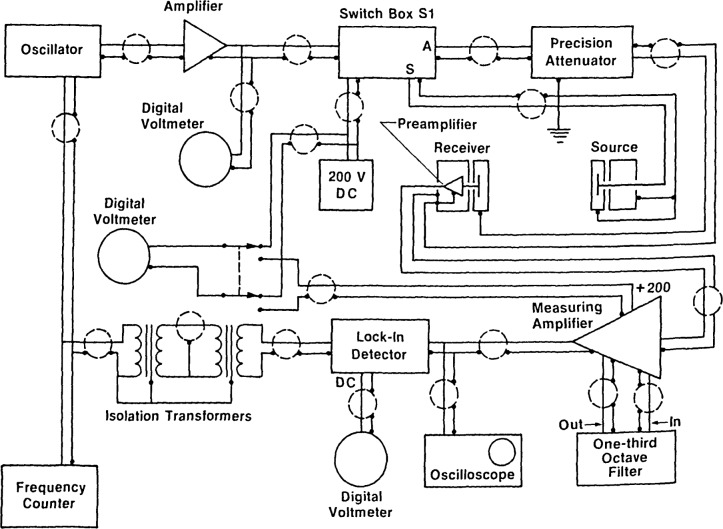
Block diagram of the equipment used for the free-field calibration of microphones.

**Figure 2:**
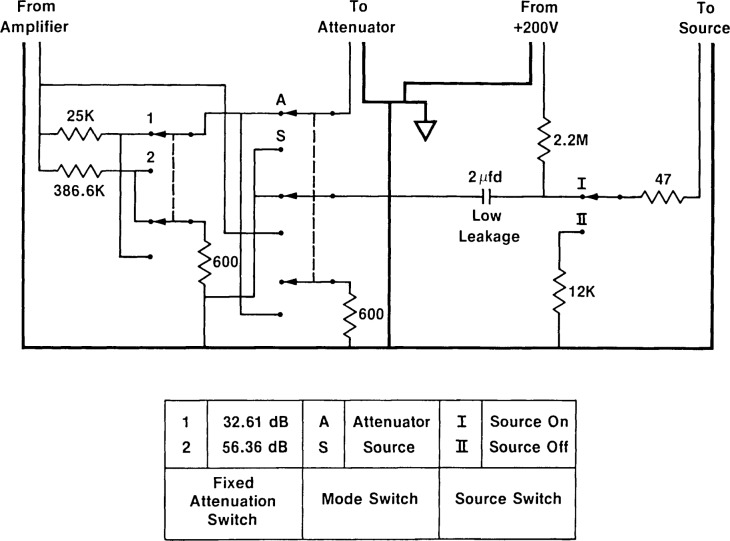
Switch box, showing nominal resistance values and measured values of attenuation.

The block diagram of the electronic instrumentation is shown in figure 1. The applied sinusoidal signal from the oscillator will take one of two paths, depending upon the setting of the switch, SI. The details of the switch are shown in figure 2. When S1 is set to position “S,” the drive signal is applied to the source microphone. The sound generated by the source microphone is picked up by the receiving microphone and preamplifier, amplified by the measuring amplifier, and then detected by the lock-in amplifier and indicated by the digital voltmeter. When the switch is set to position “A,” the signal is electrically applied through the attenuator so that the output of the attenuator acts as a voltage source in series with the electrical terminals of the receiving microphone. The electrical path following the receiving microphone remains the same. The precision attenuator, which can be incremented in steps of 0.01 dB, is adjusted so the output voltage as detected by the lock-in amplifier is the same for the two switch settings. The reading of the attenuator, therefore, expresses the ratio of the source drive voltage to the receiver microphone output voltage. This quantity is denoted *A_xy_* (*x*=a,b; *y*=a,d) in eqs (3) and (4), where *x* and *y* refer to the receiving and source microphones, respectively. Using this method, it is not necessary to know the absolute values of the drive and receiving voltages. The source switch (fig. 2) is left in position I when making measurements, and is turned to position II (Source Off) when removing or attaching the source microphone.

### 3.2 Synchronous Detector

The transmitting microphone acts as a very small single-sided electrostatic loudspeaker unit (although with a stiff diaphragm having a very high fundamental resonance frequency). Consequently, the drive voltage should be small relative to the applied dc polarizing voltage for the unit to behave as an essentially linear transducer [6], as well as to avoid ionization breakdown of the air between the diaphragm and backplate and potentially excessive stress within the diaphragm itself. The largest value of ac drive voltage used at the NBS is 10 volts rms. This limits the sound pressures, and therefore voltages, developed at the receiving microphone during calibration to rather low values, especially at the lower frequencies of calibration. For example, with many widely used 1/2-inch precision condenser microphones as both the transmitter and receiver, separated by a distance of 20 cm, the output voltage at the receiving microphone at 1500 Hz is of the order of one microvolt. The attenuation relating receiver output voltage to transmitter drive voltage is approximately 140 dB for these conditions, and signal-to-noise ratio of the measurements is a critical problem at the lower frequencies of calibration.

In order to obtain an adequate signal-to-noise ratio from the output of the receiving microphone narrow-band filters or signal averaging techniques must be used. A self-tuning lock-in amplifier makes a particularly convenient type of filter because of its narrow, but adjustable, bandwidth and its ability, when provided with a suitable reference signal phase-locked to the calibration signal, to track this calibration signal automatically.

Because of the use of the insert-voltage method to obtain accurate measurements that are relatively insensitive to many of the specific characteristics of the electronic instruments that are used in the apparatus (see sec. 2.1 of ref. [3] and the switching arrangement in fig. 1), the phases of the signals sequentially produced at the output of the receiving microphone by the driven source microphone and by the inserted attenuator signal usually will not be the same with respect to the reference signal from the oscillator. Use of the insert voltage method, however, requires that the amplified and filtered (or otherwise detected) outputs of the microphone be equal in magnitude whenever these sequentially produced microphone output signals are of equal magnitude. Consequently, for a given magnitude of input signals, the voltage magnitude detected and indicated by the lock-in amplifier must be essentially independent of the relative phase of its input and reference signals.

Some commercially-available units achieve this essential independence by shifting the phase of the reference signal to match that of the input signal. In any event, since a specification for the degree of such independence is not normally included in manufacturers’ specifications of lock-in amplifier performance, it is necessary to measure this degree of independence before using a lock-in amplifier as shown in figure 1. The lock-in amplifier used was found to have (for a given magnitude of input signal) an output independent, within 0.01 dB, of the relative phase of the input and reference signals, and was therefore considered to be a satisfactory detector for the amplified, band-pass filtered, A-weighted (to reduce low-frequency noise) output of the receiving microphone. Time constants typically ranging from 0.3 to 30 s, and occasionally to 100 s, are used when making measurements. With the longest time constant, (100 s) long-term drift in the system of figure 1 can reduce the accuracy of measurements. The time constant determines the bandwidth of the detector and, therefore, the lowest signal levels that can be measured to the desired accuracy for a given signal-to-noise ratio at the input, provided that the dynamic reserve capability of the lock-in amplifier is not exceeded (i.e., provided that this amplifier is not significantly influenced by noise or Fourier components of the input signal that lie outside the passband). The A-weight ing and one-third-octave band-pass filtering prior to the input of the lock-in amplifier eliminate harmonic Fourier components to which the lock-in detector might otherwise respond and ensure that the lock-in amplifier is operated within its dynamic reserve capability.

### 3.3 Grounding

Although the grounding procedures used are straightforward, they must be carefully followed since the magnitude of the crosstalk voltage must be less than 0.1 percent of the signal voltage magnitude for the error in signal measurement to be less than 0.01 dB. A seemingly slight variation in the grounding procedures can produce a crosstalk level that is higher than the desired signal. The ground connections and signal leads are shown in figure 1. The ground is carried through by only one path. A problem arises because the lock-in amplifier has two inputs, one for the signal and one for the reference. The signal path to the reference channel, therefore, uses two audio-frequency transformers in cascade for isolation of the ground. (One transformer did not give sufficient isolation, presumably because of capacitive feedthrough.) Each piece of equipment has the power ground broken by means of three-pin to two-pin adaptors at the connection to the power line. For considerations of safety, the entire system is grounded to earth at the attenuator panel, although connecting this ground makes no difference in the crosstalk level.

### 3.4 Preamplifier and Microphone Ground Shield

**Figure 3:**
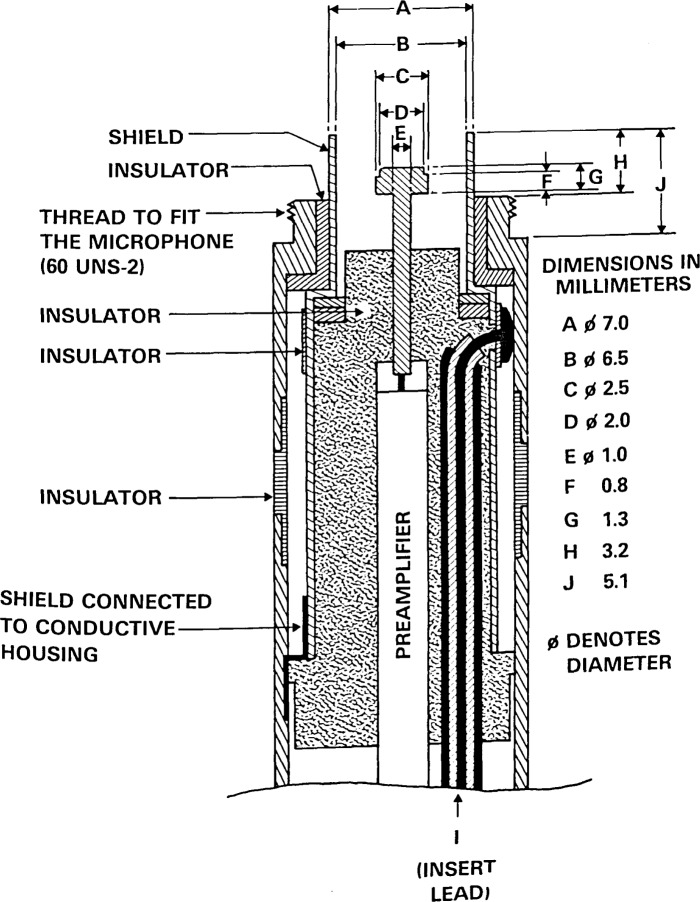
Cross section of the preamplifier to which the receiving microphone is attached, and the assembly on which the transmitting microphone is mounted, showing ground-shield and other key dimensions. The dimensions A through J are the same for the preamplifier and transmitting microphone assembly. In the transmitter, the preamplifier is replaced by a shielded lead to the center contact pin.

The preamplifier is a modified version of a commercially-available device (Bruel and Kjaer Type 2619)^2^ commonly used with 1/2-inch microphones. The heater has been removed to prevent thermal gradients and atmospheric convection from influencing either the microphone or the sound transmission path between transmitter and receiver, and thus affecting the measurements. The center connector shield has been grounded, and a plastic insulating ring has been installed so that the microphone cartridge body shell can be connected to a shielded lead that is used to provide the insert voltage. The geometry of the center pin shield has not not been changed. Figure 3 shows the ground-shield and other key dimensions of the modified preamplifier without electronics. The preamplifier is coaxially mounted on a hollow rod of 12.7 mm (1/2-in) external diameter.

### 3.5 Transmitter

The transmitter to which the source microphone is connected is mechanically identical (with regard to key dimensions A through J) to the preamplifier. Instead of the preamplifier, a shielded lead is connected to the center pin that contacts the center conductor of the microphone. The electrical connection to the microphone body is effected by means of a shielded lead so the driving-point electrical impedance of the microphone can be measured *in situ*. Figure 3 shows the key dimensions of the transmitter assembly. The transmitter is also coaxially mounted on a hollow rod of 12.7 mm diameter.

### 3.6 Mounting

The rods supporting the preamplifier and transmitter are passed through bearings on opposite walls of a small anechoic chamber. The rod supporting the transmitter is normally fixed in one position. The rod supporting the preamplifier is attached to a screw-driven mechanical slide with a line-scale position indicator, external to the chamber. This arrangement allows the receiving microphone to be placed at any desired distance up to 31 cm from the source microphone, with a repeatability in position of about 0.1 mm.

### 3.7 Anechoic Chamber

The tests are performed in a chamber with a width of 2.1 m, a height of 1.6 m and a depth of 1.6 m between wedge tips (free volume of 5.4 m^3^). The fiberglass wedges are 0.3 m deep. The chamber is single-walled but the use of a one-third-octave band-pass filter, A-frequency weighting in the measuring amplifier, and a lock-in amplifier for the detection of the signal provides adequate rejection of ambient noise. The chamber is located in a quiet, windowless control room that is separated from the rest of the NBS Sound Building by reinforced concrete walls approximately one foot (0.3 m) thick. This room is located in the most quiet part of the building, as far as possible from mechanical HVAC equipment at the opposite end of the building. The chamber rests on elastomeric blocks on the floor of this control room; these blocks provide vibration isolation from the floor.

## 4. Acoustic Center

**Figure 4:**
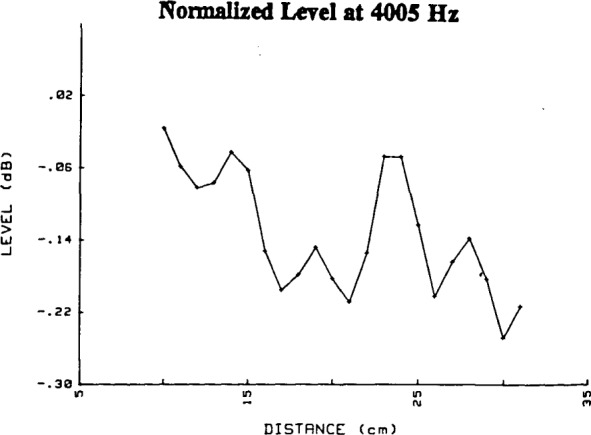
Deviations in level from an inverse distance relationship for the normalized (with respect to grid-to-grid separation distance) and corrected (to remove effects of atmospheric absorption of sound) data of two high-sensitivity, e.g., −26 dB re 1V/Pa, 1/2-inch microphones at 4005 Hz. The abscissa is grid-to-grid separation distance.

The virtual locations of the source and receiving microphones are usually not at the diaphragms of the microphones but at some nearby positions known as the acoustic centers. No general analytic procedure exists for the determination of the location of the acoustic centers. An empirical method is used in this paper, based on [3] and [5]. At low frequencies and at distances commonly used in free-field calibration by the reciprocity method, these positions are in front of the diaphragm of each microphone, i.e., outside its exterior surface. A determination of the positions of the acoustic centers was obtained using the inverse relationship between the amplitude of the sound pressure at the receiving microphone and the distance between the acoustic centers of the source and receiving microphones. Data were obtained by holding the source microphone at a fixed position and varying the position of the receiving microphone for grid-to-grid separations ranging from 1 cm to 31 cm. The attenuator settings in decibels for a specified drive voltage were recorded and thus constituted relative levels at the receiving microphone. Each level was converted to relative amplitude and a correction was made for the absorption of sound by the atmosphere [7]. In order to account for the inverse relation between amplitude and grid-to-grid spacing, the amplitude for each position was normalized by multiplying it by the grid-to-grid spacing for that position. If the correction for atmospheric absorption were exact, if the acoustic centers were at the grid of each microphone, if the microphones had been within each other’s far field only (i.e., with no near-field component, however small), and if the acoustical environment had been perfectly anechoic, then the normalized amplitudes for a given frequency would all have the same value. In the discussion which follows, only data from separations of 10 cm to 31 cm are considered, so that amplitude values only from positions at which the sound pressure was dominated by the far field of the microphones are used (it does not necessarily follow, however, that the near-field component was negligible at all frequencies). For purposes of presentation, the normalized data, shown in figure 4 at a typical frequency of 4005 Hz, are converted back to level. These values of level are not identical, although the differences are not large.

The normalized amplitude data were then evaluated by a first-order polynomial regression procedure which performed a least-squares fit of a straight line to the data shown in figure 4. This fit was done on a desktop computer (Hewlett-Packard 9836C, using regression analysis software contained in HP part no. 98820-13618). Had the above conditions been met, and had the acoustic centers been at the microphone grids, the data set would have a slope of value zero, and the coefficient of the first order term in the polynomial regression would have been zero. Such was not the case. The constant term and the first-order coefficient produced by the regression for 4005 Hz are:

$$\begin{array}{l} \text{constant term}=0.20113\;\text{db}\\ 1\text{st order coefficient}=-0.00015\;\text{db}/\text{cm} \end{array}$$

Such slight departures from zero slope are also typical of the data at frequencies other than 4005 Hz.

The distance corrections due to the virtual locations (apparent acoustic center positions) of the source and receiving microphones were determined and incorporated into the relative amplitude data (corrected as before for atmospheric absorption of sound) at each frequency by:

1. multiplying each relative amplitude datum obtained at the given frequency by (*d* + Δ*d*), where *d* is the physical separation between microphone grids (grid-to-grid distance) for that datum and Δ*d* is a trial correction for the spacing between microphones due to the apparent acoustic center positions at that frequency.
2. replotting on a CRT display each datum above with abscissa (*d* + Δ*d*) and reapplying the first-order polynomial regression to the data for this frequency and,
3. iterating steps 1) and 2) above on the desktop computer for different values of Δ*d* until the value of the first order coefficient (and consequently the slope of the plotted line) was essentially zero. The value of Δ*d* corresponding to this zero slope condition was then considered a measure of the distance correction at that frequency for the range of distances represented in the data. The data corresponding to this zero slope condition were then renormalized by division by the value of the constant term in the regression corresponding to this zero slope condition, and consequently also corresponding to the final distance correction. These renormalized data are hereinafter referred to as the “renormalized amplitude data,” and, when converted to level and plotted, as the “renormalized data converted to level,” the “renormalized data,” or the “level renormalized for measured grid-to-grid correction.”

Since two microphones of the same type were used as the transmitter and receiver, the displacement of the acoustic center from the grid is considered to be one-half the amount of the distance correction, with a negative value indicating the displacement is away from the microphone; i.e., outside the exterior surface of its protection grid.

The corrections for the spacing between the two microphones at various frequencies are shown in the second column of table 1. In order to obtain distance corrections which are a smoother function of frequency than these (hereinafter referred to as the “measured corrections”) obtained directly from the measured data, these corrections are themselves subjected to a second-order polynomial regression process. The resulting smoothed values (hereinafter referred to as the ‘‘second-order fit corrections”) are shown in the third column of table 1. Table 1 also shows the resulting calculated changes in level at two specific distances which occur because the acoustic center is not at the grid of the microphones. Specifically, the fourth column gives the changes which would occur for a grid-to-grid spacing of 20 cm if the acoustic centers are assumed to be at the positions given by the measured corrections, rather than at the microphone grids. The fifth column gives the changes in level which would occur if the acoustic centers are assumed to be at the positions given by the second-order fit corrections. The last columns repeat these calculations for a grid-to-grid spacing of 15 cm.

**Figure 5-18:**
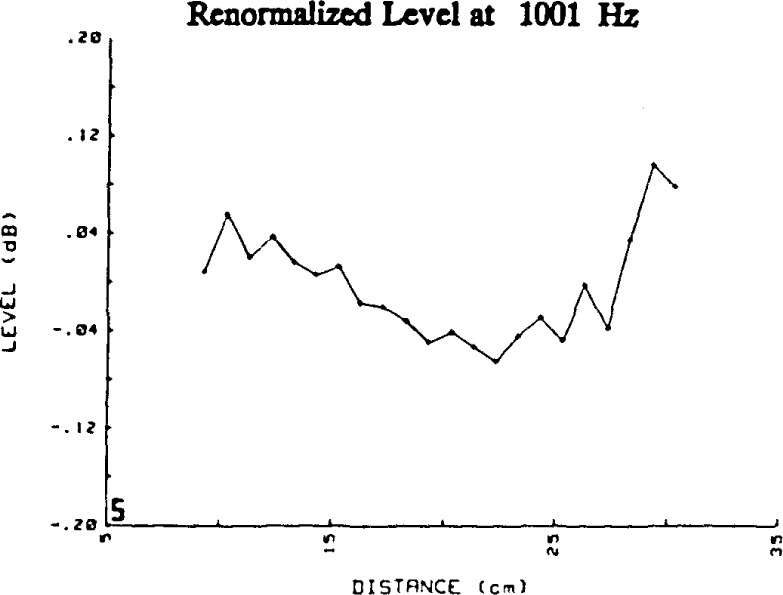

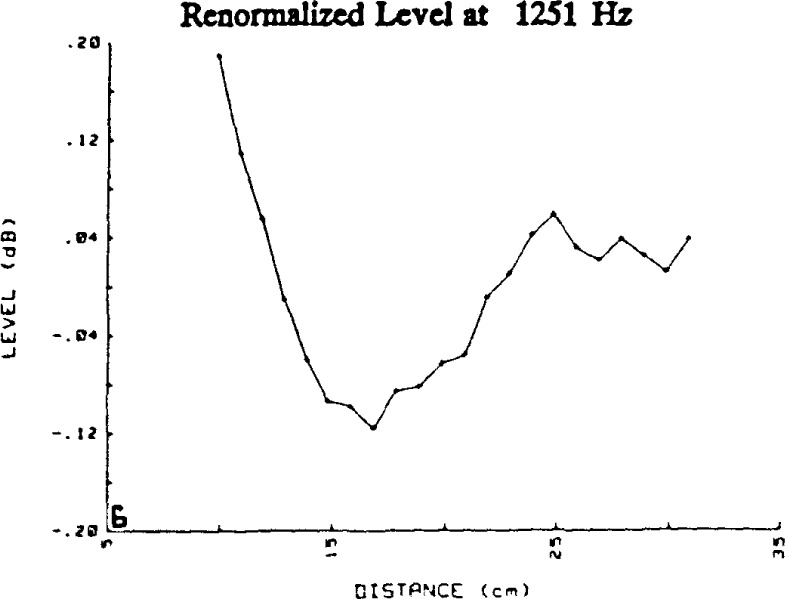

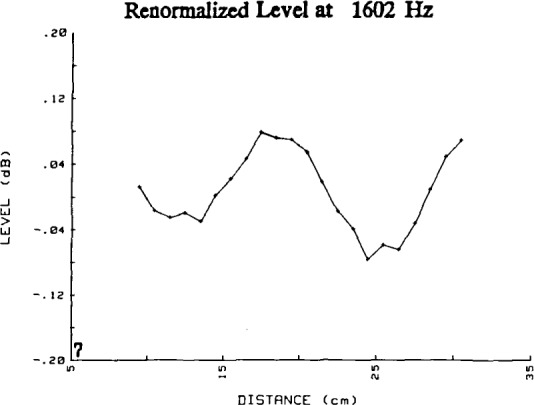

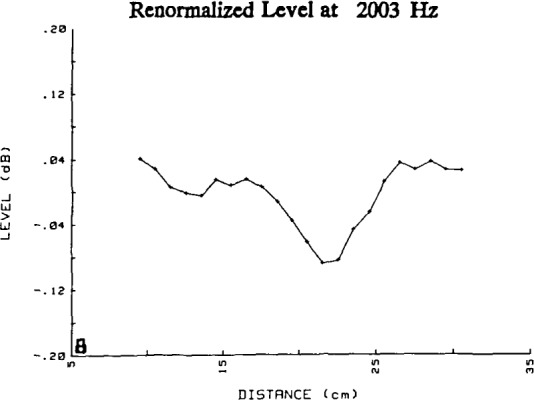

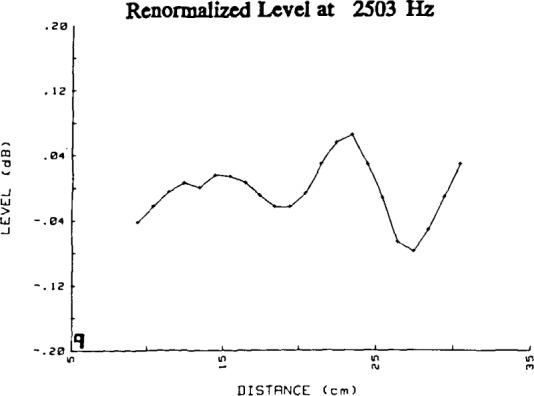

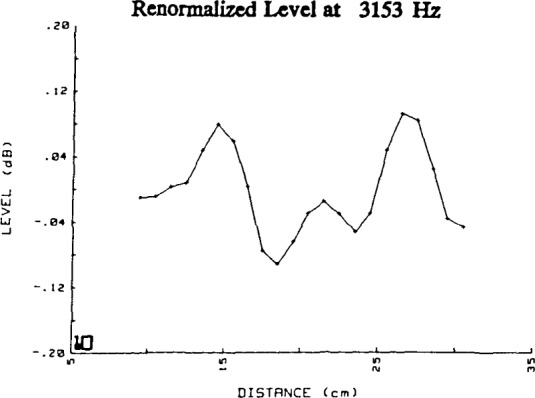

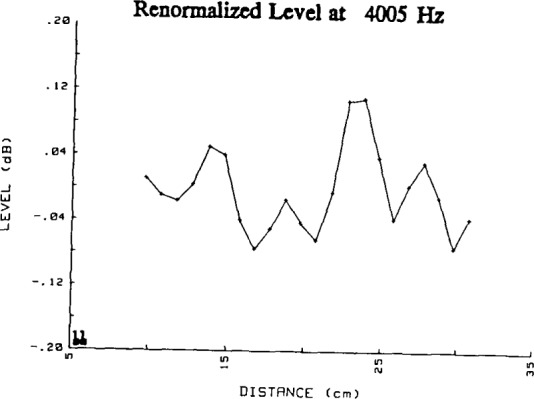

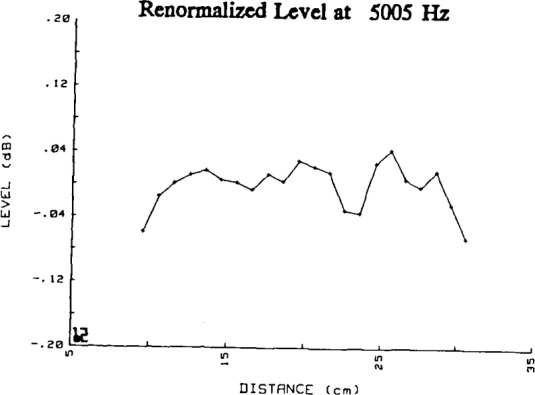

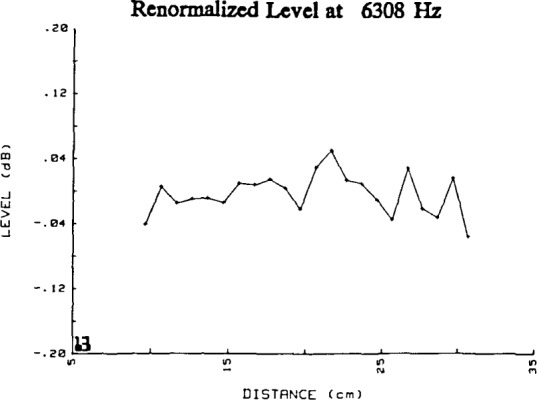

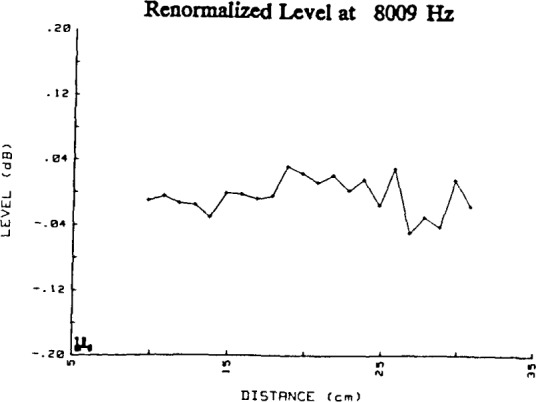

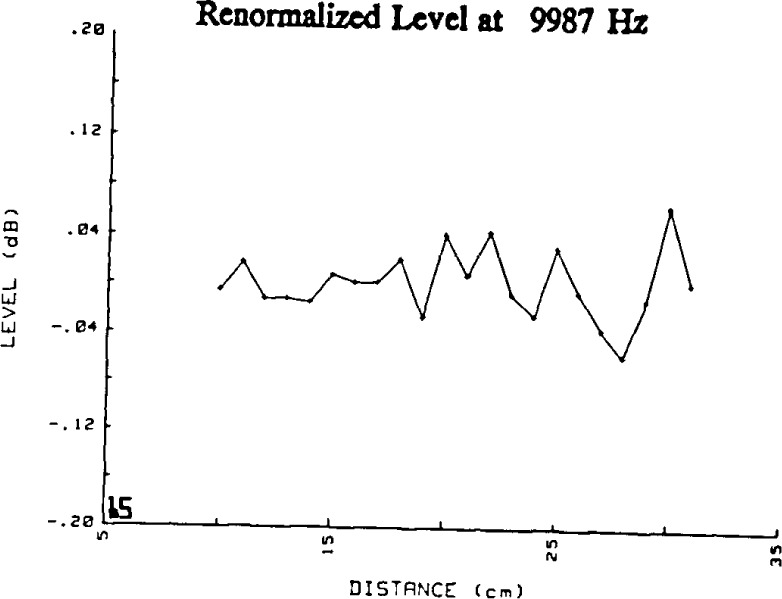

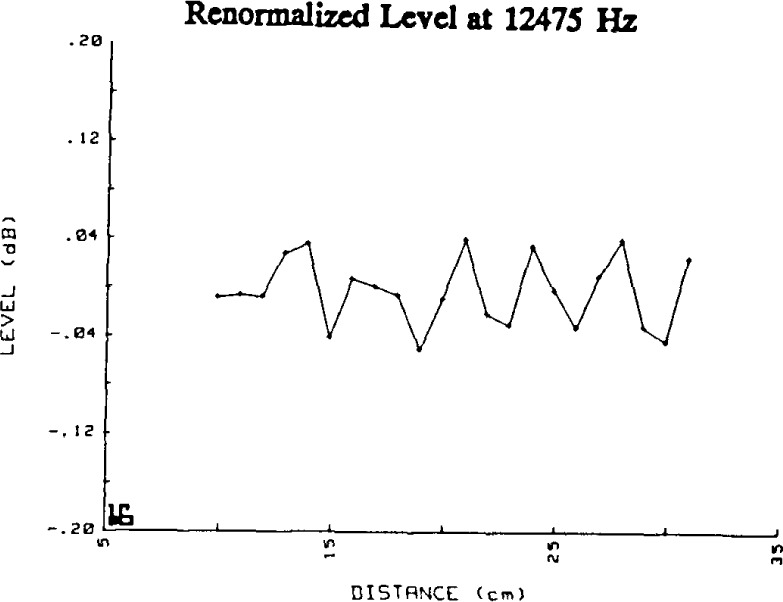

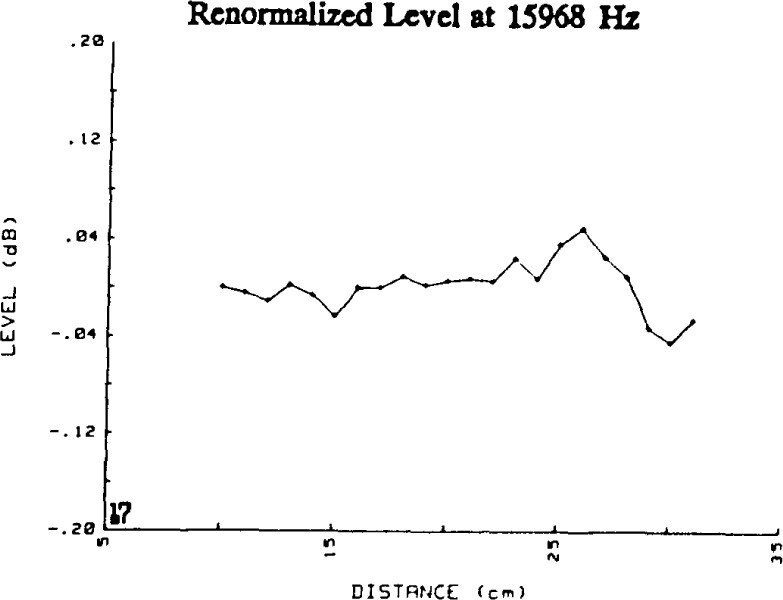

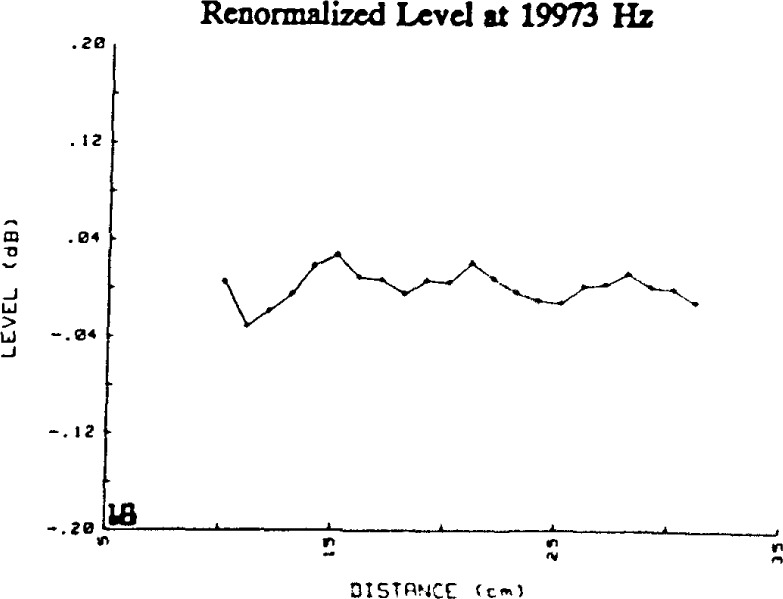
Deviations in level from an inverse distance relationship for data such as those in figure 4, but renormalized (with respect to separation distance corrected for the calculated acoustic center positions). Each figure shows data at a different frequency in the range from 1 kHz to 20 kHz. All data shown are from high-sensitivity 1/2-inch microphones. The abscissa is the sum of the grid-to-grid separation distance and the measured grid-to-grid acoustic center correction.

Figures 5–18. show the plots over the range 1 kHz–20 kHz for the renormalized data with the amplitudes converted to level. Clearly, even though the first-order regression coefficient is es sentially zero, not all renormalized data points show the same level. Table 2 summarizes these renormalized levels at 15 cm, 20 cm and at the distance between 15 and 25 cm at which the worst deviation of measured level from the zero value occurs, and the value of that deviation. These deviations from zero give insight into the spatial characteristics of the sound pressure field in the chamber, and the proper choice of test parameters. These deviations most probably could be due to unaccounted-for near-field effects, or one or more of three factors: normal modes (standing waves) due to reflections within the chamber, standing waves due to reflections between the microphones, and electrical crosstalk. We will consider the possible influence of these three factors in sequence.

Factor 1. Many of the reflections within the anechoic chamber will produce deviations from the inverse distance relationship which appear somewhat random. However, in the simplest example, axial modes in the chamber that include waves with propagation vectors parallel to the axis of rotational symmetry of the microphone will produce pressure maxima or minima at integer multiples of one-half wavelength apart. For example, figure 10 shows maxima at separation distances of about 15 and 27 cm. The differences of 12 cm in the separa tion distances correspond reasonably closely to the wavelength for 3.15 kHz, which is approximately 11 cm. However, there is no maximum of closely comparable value at about 20 cm to 21 cm, so that the pressure standing wave pattern is clearly influenced by other (non-axial) modes within the chamber, or by other factors, as well.

Factor 2. Standing waves due to reflections between the microphones themselves will also produce maxima or minima at separation distances that are integer multiples of one-half wavelength. Such effects are seen at some frequencies. For example, at 4.0 kHz the minima seen in figure 11 are at separation distances of 12, 17, 21, 26, and 30 cm. The wavelength at 4.0 kHz is approximately 8.6 cm. However, it is not possible completely to separate the effects which are due to standing waves caused by reflections from interior surfaces of the chamber (or “room reflections”) and those due to standing waves caused by reflections between the microphones. It would be expected that the effect of microphone reflections would become more pronounced as the frequency is increased (as wavelengths become comparable to the microphone diameter), and less pronounced as the separation distance between the microphones increased, since the microphones do become much more directional at high frequencies, but sound is not scattered from the microphones by specular reflection in the range of frequencies studied. It is also expected that the effect of room reflections would be more pronounced at the lower frequencies of measurement, at which the dimensions of the sound-absorbing wedges on the walls are smaller compared to the wavelength of the sound. Since the observed deviations in level become larger at lower frequencies, it would appear that room reflections are more significant in effect than standing waves due to reflections between the microphones. Although the effects of such standing waves do not appear important in the current system, these effects most probably can be reduced even further or eliminated by tilting the plane of either microphone diaphragm a few degrees. However, such a controlled shift in our chamber would require extensive modifications to the microphone-supporting structure.

**Figure 19:**
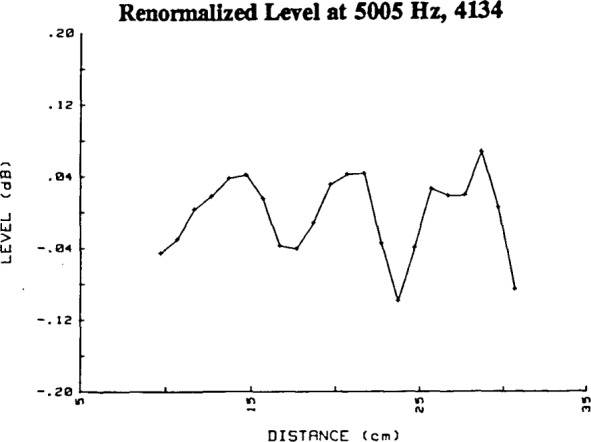
Deviations in level from an inverse distance relationship for the renormalized (as in figs. 5–18) data from two conventional, e.g., sensitivity about −38 dB re 1V/Pa, 1/2-inch microphones for a frequency of 5 kHz.

Factor 3. If present, significant crosstalk between the drive signal and the detection system will produce relative maxima or minima spaced one wavelength apart. Such phenomena do appear at some frequencies, as can be seen by comparing figures 12 and 19 for 5005 Hz. The somewhat greater deviations in the data for conventional (lower sensitivity) microphones (Bruel & Kjaer Type 4134) when compared to the data for high-sensitivity microphones (Bruel & Kjaer Type 4165) could indicate some degree of crosstalk. Fortunately, it was possible to reduce the system crosstalk by an additional 10 dB after this greater deviation for conventional microphones was noted. The deviation was not reduced by the decrease in crosstalk. The larger deviation in the data for these low-sensitivity microphones remains unexplained, but may be due to the greater uncertainty of the measurement of the lower output levels, to unknown changes in the standing wave pattern in the chamber associated with slight uncontrolled changes in ambient environmental conditions, or to unwanted sound produced by the stimulus-generating apparatus and therefore correlated with the signal.

Ideally, the length of the traverse used to determine position of the acoustic center should be an integral number of wavelengths in order to minimize biasing of the data from end effects. Below 1.6 kHz, the traverse in the far-field is necessarily less than one wavelength, since it is not possible to obtain data at an adequate signal level at separations of more than 31 cm at low frequencies. This limitation on the length of the traverse may explain the apparent decrease in the magnitude of the measured grid-to-grid correction for the acoustic centers (col. 2 of table 1) at 1.25 kHz. It may be possible to slightly increase the accuracy of the evaluation of the acoustic center position at frequencies higher than 1.6 kHz by truncating the data from traverses at an appropriate distance. This approach is under consideration.

Another assumption in the use of the data remains to be tested, namely that a grid-to-grid spacing of 10 cm produces far-field conditions. This can be checked by noting whether the slope of the renormalized data is zero at the 10 cm spacing. Deviations in the slope due to chamber effects such as standing waves make it difficult to determine if the slope is indeed zero at this distance. An inspection of the data does indicate that spacings of less than this amount could not be used. This is another area in which slight improvements in accuracy may be possible in the future.

**Figure 20:**
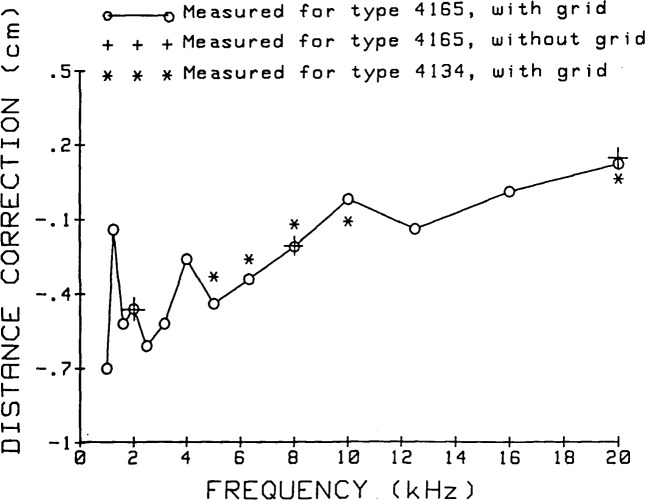
Summary of measured acoustic-center-position corrections for two 1/2-inch microphone types from 1 kHz to 20 kHz. The ordinate shows the distance correction for two microphones, to be added to the grid-to-grid separation of the microphones. One set of measurements for Type 4165 microphones was taken with their protection grids removed; these distance corrections are to be added to the grid-to-grid separation that would apply if the grids were in position on the microphones.

Most of the above determinations were made with Bruel & Kjaer Type 4165 microphones, with the protective grids in place. Essentially similar measurements were performed at a limited number of frequencies with the grids removed from the microphones, and for other types of 1/2-inch microphones. Very long time constants and time-consuming mechanical adjustments can be required for these measurements. In fact, it is not even feasible to make such measurements over the lowest portion of the frequency range with other than high-sensitivity microphones. Figure 20 summarizes the results for all types of microphones examined. At three frequencies spanning the frequency range, measurements were made using Type 4165 microphones with the grids removed. (For this condition, distance measurements were made from the position which the grid occupies when it is in place.) The effect of the grid upon the distance correction is seen to be negligible. The results for the Type 4134 microphone do show slight differences from the Type 4165 results. However, this difference is small compared to the total uncertainty of measurement. Furthermore, the higher signal level produces more accurate data for the Type 4165 microphones, and the only significant dimensional differences between the Types 4165 and 4134 microphones are differences in length which, for a given distance from the microphone grid position, should not affect the relative acoustic center location. Consequently, the data obtained with the 4165 microphones with grids in place are used for all calibrations of 1/2-inch microphones.

## 5. Uncertainties in Determination Of Microphone Response Level

An inspection of eqs (2) and (3) shows the principal sources of uncertainty in the determination of *M*_fa_ and *R*_fa_ (uncertainties for *M*_fb_ and *R*_fb_ are similar to those in *M*_fa_ and *R*_fa_). Note that, because of the square root appearing on the right-hand side of eq (2), most of the terms that will occur in the evaluation of eq (3) are either divided by two or are expressed as 10 times the logarithm of some quantity, instead of 20 times the logarithm of the quantity. This means that an uncertainty of, for example, 0.01 dB in an attenuator setting produces a calibration uncertainty of only 0.005 dB.

In the expression [*A*_bd_–*A*_ad_–*A*_ba_]/2, the uncertainty is considered random, and is dominated by system noise and drift, except possibly at the very highest frequencies of calibration where attenuator inaccuracy (less than 0.01 dB in each measurement *A*_xy_) makes a significant contribution. Expressed as two standard deviations (as are all random uncertainties described hereinafter unless otherwise noted), the uncertainty in the above expression may be as large as 0.03 dB at frequencies from 5 kHz to 20 kHz, and as large as 0.09 dB at frequencies from the lower calibration limit of 1 kHz to 5 kHz for high-sensitivity 1/2-inch microphones, and from 1.25 kHz to 5 kHz for low-sensitivity 1/2-inch microphones.

The frequency in the eq (5) is raised to the second power, and consequently the term 20 log *f* will appear when eq (3) is evaluated. In the current system using a quartz-stabilized frequency synthesizer, the frequency can be determined with high accuracy, to within 20 parts per million or better, resulting in an uncertainty of .0002 dB, which is considered random.

The barometric pressure and the temperature each can be measured to an accuracy which is better than one part in one thousand. An uncertainty of one part in one thousand in each of these quantities produces a calibration uncertainty of 0.004 dB in each. This uncertainty is considered random for both quantities. It is estimated from work done with the NBS system for the pressure calibration of microphones that the uncertainty in the determination of 10 log_10_*C*_a_ contains systematic and random components of uncertainty that are each less than 0.005 dB.

The term 4.343 *α r*_ab_ is itself small, the maximum value being 0.07 dB at 20 kHz. An uncertainty of 10 percent for the product of *α r*_ab_ will produce an uncertainty in the calibration which is less than 0.007 dB, and is considered random.

Factors affecting the determination of the acoustic center have been discussed in section 4. Some idea of the uncertainty of that determination may be obtained by using the data in tables 1 and 2. A grid-to-grid spacing of 20 cm is chosen for calibration purposes.

The differences between the effects of the second-order-polynomial-fit corrections for distance and the effects of the measured corrections are taken to represent the influences of the uncertainties in the determination of the distance. To the influence of each of these uncertainties expressed as an effect upon corrected level at each frequency is added the corresponding worst-case-distance variation in level shown in column 5, table 2. The worst-case situation for the resulting sum then occurs at 4.005 kHz, where this sum shows a possible discrepancy of magnitude 0.17 dB, for a calibration uncertainty of 0.085 dB. An estimate of 0.1 dB is then a conservative estimate for the magnitude of credible bounds on the calibration uncertainty attributable to the determination of the effective distance between acoustic centers at frequencies 1 kHz to 5 kHz. Of this value, 0.06 dB is considered systematic, and 0.04 dB is considered random. At frequencies above 5 kHz up to 20 kHz, this uncertainty is considered to have a magnitude of credible bounds of 0.055 dB, which is the sum of a systematic component of 0.015 dB, and a random component of 0.04 dB.

**Figure 21:**
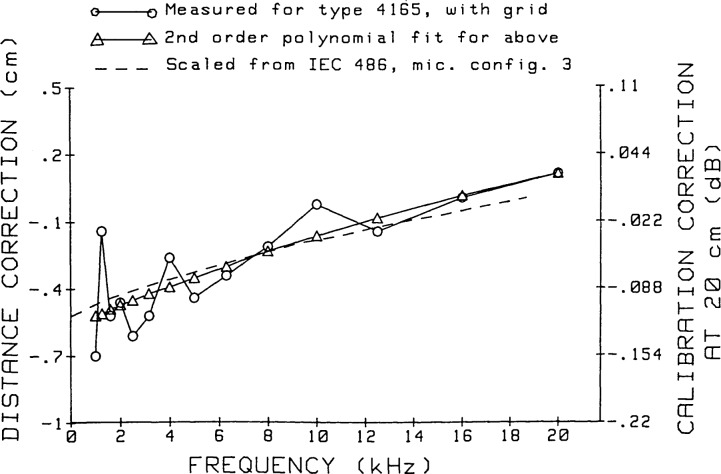
Summary of acoustic-center-position correction for Type 4165 microphones. Two scales for the ordinate are given: the left scale shows the corrections for two microphones, to be added to the grid-to-grid separation of the microphones; the right scale shows the effect upon calibrated microphone response level of applying these corrections to a measured grid-to-grid separation of 20 cm.

The values obtained from the second-order polynomial fit are checked further by comparing them (fig. 21) to the corrections for the acoustic center locations given in figure 6 of reference [5] for a microphone with grid. In order to compare the distance corrections for two microphones, these corrections from reference [5] for the positions of the acoustic center are doubled. They are then divided by 1.87, which is the ratio of the diameters of the nominal “1-inch” microphones and 1/2-inch microphones. The frequency scale is multiplied by 1.87. These corrections give excellent agreement with the corrections obtained from the polynomial fit, the calibration differences attributable to the differences in these corrections for a spacing of 20 cm being no more than 0.02 dB at any frequency.

It might appear that the accuracy could be improved by correcting for the measured deviations at a specific distance and frequency. In general, this is not the case. The data were taken using an oscillator which had low distortion and high stability in both amplitude and frequency. However, it could not be set precisely and conveniently to the desired one-third octave center frequencies. Furthermore, calibrations may be needed at other than the one-third octave center frequencies. A frequency synthesizer is now used in the system instead of the oscillator, and its frequency can be set precisely and conveniently to the desired values. Even so, changes in atmospheric conditions or in the configuration of the chamber will cause changes in the standing wave patterns, especially at high frequencies. For these reasons, the results should be considered exemplary, rather than definitive for all conditions.

The second-order polynomial fit corrections are the ones actually used for calibration purposes. Such use appears strongly justified by the close agreement between the scaled values from reference [5] and the second-order polynomial fit to our measured correction values for microphone acoustic center positions.

Although each determination of the position of the acoustic center contains uncertainties because of the reasons just discussed, the calibration accuracy is not degraded to a similar degree. The correction for the displacement of the acoustic center is a small fraction of the total spacing between the microphone diaphragms, as a consequence of our deliberate choice to work at relatively large (most typically, about 20 cm) spacings.

The purpose of the present study has not been to establish the exact positions of the acoustic centers, but to determine the corrections with sufficient accuracy to perform the calibration. Great effort to achieve adequate signal-to-noise ratios and low crosstalk has enabled measurements to be performed at the relatively large separation distance of 20 cm. This separation distance has also been selected since at all the test frequencies shown in table 2 the value of the renormalized level in column 3 is in magnitude 0.07 dB or less, a smaller value than the corresponding magnitude for either the renormalized levels at 15 cm (column 4) or the worst-case renormalized levels (column 5).

Table 3 shows the estimated random uncertainty in decibels of each term from eqs (3) and (5). Also shown are the random uncertainties (in decibels) contributed to a microphone response level by sources not apparent in eqs (3) and (5): determination of the polarizing voltage, and departures of about 0.2 degree from the normal incidence specified for calibration. These departures reflect the tolerances on mechanical alignment of the rods upon which the microphones are mounted. Table 4 shows estimated credible bounds on the significant systematic components of uncertainty. We have assumed that the standard deviation for each term is one-half the amount shown in table 3. Also, we consider that the uncertainties in table 3 are independent. Then, the overall rootsum-square random component of error (assumed to be two standard deviations) of the measurement ranges from 0.05 dB at high frequencies to 0.10 dB at low frequencies, as shown in table 5. The overall systematic component of uncertainty, expressed as the sum of the systematic components, ranges from 0.02 dB at high frequencies to 0.06 dB at low frequencies. Our initial objective in developing this measurement service was an overall measurement uncertainty of 0.2 dB or less. Thus, at all frequencies, the sum of the systematic error and two standard deviations, which sum is 0.16 dB at low frequencies and 0.07 dB above 5 kHz, is well within the initial objective.

As mentioned in section 2, the accuracy can be improved by taking additional sets of measurements, a procedure which reduces random uncertainty but does not alter the worst-case estimate of systematic uncertainty. The fixed-cost calibration service from NBS consists of one set of measurements, although a second set or more can be taken at additional cost upon request.

Any significant increase in accuracy of calibration at frequencies from 1 kHz to 5 kHz would require that the signal-to-noise ratio of the detection system be increased. Such an increase would require that either the preamplifier electrical noise be reduced or that the detection time be increased, which in turn would require a decrease in the longterm (during insert voltage measurement) drift of the detection system. To increase the accuracy at all frequencies, the distance which corresponds to the spacing between the apparent acoustic centers would have to be known more accurately, which would require a more nearly perfect anechoic environment.

While further refinement of apparatus is not only possible, but will ultimately become necessary to meet evolving needs, the uncertainties in the current NBS fixed-cost calibration services for 1/2-inch microphones in their frequency range 2.5 kHz to 20 kHz represent an improvement upon those in the corresponding previous (now discontinued) NBS services for “1-inch” laboratory standard microphones in 1965 [8]. When uncertainty is comparably expressed for the “1-inch” microphones as two standard deviations plus the estimated bounds on systematic error, the uncertainty in the calibration of “1-inch” microphones ranged from 0.16 dB to 0.44 dB over this frequency range.

The frequently lower transmitting and receiving sensitivities and the higher electrical impedances of the 1/2-inch microphones result in poorer signal-to-noise ratios, larger crosstalk, and consequently much more formidable difficulties in calibration than those encountered with “1-inch” microphones. Nevertheless, the superiority of current instrumentation, apparatus, and procedures has enabled lower uncertainties to be achieved in the free-field calibration of 1/2-inch microphones than had been achieved in 1965 for “1-inch” microphones.

Emphasis has been given to services for free-field calibration of 1/2-inch microphones because the use of these microphones to perform precision practical measurements is usually not nearly as limited by diffraction effects as is the use of “1-inch” microphones. While “1-inch” laboratory standard microphones can be exceptionally accurate and stable instruments for the measurement of well-specified sound fields under laboratory conditions, such as a uniform plane wave of known angle of incidence, or the sound pressure in an acoustic coupler, practical measurement situations often involve sound fields that are not so well specified. It may be difficult or impossible to choose a single, well-defined calibration (free-field at specified angle of incidence, pressure, or random incidence) that is applicable. The “1-inch” standard microphones are sufficiently large that diffraction effects at frequencies well within the audible range cause substantial differences among the available calibration choices, and ambiguity in choice of the applicable calibration can result in large uncertainty in measurement. The difference between free-field (normal incidence) and pressure response levels for a “1-inch” microphone increases as frequency is increased from a few hundred Hz to about 12 kHz, and can be as large as 9 dB at frequencies below 10 kHz (ref. [3], fig. A3). This difference is about two orders of magnitude larger than the typical uncertainty in reciprocity calibration at these frequencies. Consequently, if “1-inch” microphones are used for measurement of sounds having appreciable spectral energy at high frequencies, overall measurement uncertainty can dwarf uncertainties due to calibration.

Use of 1/2-inch microphones can reduce these uncertainties. Calibrations by reciprocity performed at NBS upon 1/2-inch microphones under comparable conditions have shown that the difference between free-field (normal incidence) and pressure response levels is less than about 4 to 5 dB (depending upon microphone type) at frequencies below 10 kHz, and reaches 9 dB only at about 19 to 20 kHz; i.e., the region of large diffraction effects is shifted to frequencies above 10 to 15 kHz. This shift increases the accuracy of most commonly-per formed measurements for two reasons:

1. The objective in many practical situations is to measure the broad-band sound pressure level weighted by one of the standard A, B, or C frequency-response weighting characteristics [9]. These characteristics provide appreciable attenuation at frequencies above 10 to 15 kHz, reducing effects of measurement uncertainty at such high frequencies on the measured broad-band level.
2. The spectra of most common sounds tend to decrease in amplitude as frequency is increased beyond several kHz, so that relatively little spectral energy is likely to be present at frequencies where diffraction effects are large. Consequently, the lesser uncertainty associated with diffraction effects for 1/2-inch microphones at frequencies below 10 to 15 kHz will usually contribute to more accurate measurement of broad-band levels and to more accurate spectral energy data.

## 6. “One-Inch” Microphones

One-inch microphones can be calibrated by placing commercially-available “1-inch” adaptors on the 1/2-inch mounting hardware. This arrangement produces two nonstandard test conditions. The mounting rod for the microphone is not constant in diameter, but necessarily tapers from a diameter of 0.936 inch at the microphone down to 1/2 inch. The ground-shield geometry is not that specified for calibrating “1-inch” microphones [3], but is that used for 1/2-inch microphones (fig. 3). However, both of these conditions are often encountered in the actual use of some “1-inch” microphones. Under these conditions, the current instrumentation should also result in improved (relative to the 1965 values) values of uncertainty in the calibration of “1-inch” microphones.

Western Electric 640AA “1-inch” microphones cannot be tested with this mounting as the shield of the 1/2-inch preamplifier contacts the center connector of the microphone. Rods and mounting hardware for the present system are available for such “1-inch” microphones. However, the time required for removing the 1/2-inch rods, mounting and aligning the items for these “1-inch” microphones, and ultimately replacing and realigning the 1/2-inch rods is such that a relatively inexpensive fixed-cost calibration service for such “1-inch” microphones is not offered currently.

During calibrations at the NBS, the microphone denoted (d) in eqs (3) and (4) is usually of the 1/2-inch type. However, it could be a “1-inch” microphone, since its purpose is to act as a source in sequential measurements determining the ratio of sensitivities of the two microphones (denoted (a) and (b)) being calibrated. During these calibrations the microphones (a) and (b) are both 1/2-inch microphones, or are both “1-inch” microphones with identical mounting adapters, so that microphones (a) and (b) are substantially similar in size and directional pattern. This similarity helps to ensure that the sound pressure sequentially produced at microphones (a) and (b) by the source microphone (d) is essentially the same, even though the test chamber is not perfectly anechoic. Consequently, the effects of this potential source of additional uncertainty are prevented.

## 7. Comparison of Experimental and Theoretical Plane-Wave Free-Field Corrections for One-Half Inch Microphones

The mounting of the microphones on 1/2-inch diameter rods extending through the walls of the anechoic chamber during free-field calibrations at normal incidence by the reciprocity method closely approximates the geometry of mounting the microphones upon semi-infinite rods of the same diameter. This choice of a uniform mounting geometry is deliberate, because use of various mounting geometries with the same microphone cartridge would cause differences in its measured free-field response levels. Such differences are due to diffraction about the microphone and its supporting structures, and occur at frequencies sufficiently high that a wavelength of sound is not large compared to the microphone and significant dimensions (e.g., rod diameter) of its supporting structure.

Effects of diffraction upon the sensitivity of practical microphones constitute a principal reason for performing free-field calibrations: if a microphone of adequate sensitivity for a measurement were of negligibly small dimensions relative to the range of wavelengths of sound to be measured, the pressure and the free-field response levels of the microphone would be essentially identical for such a measurement.

Unfortunately, this is not the case for 1/2-inch (or other commonly used) microphones. Furthermore, for a given microphone it is not practical at all frequencies of interest to calculate the plane-wave free-field correction, defined in reference [3] as the free-field response level at a given frequency and orientation with respect to the direction of sound propagation, minus the pressure-response level. This impracticality occurs because this calculation is influenced by the relation between the radiation impedance loading the microphone diaphragm and the acoustic impedance of the microphone itself at that diaphragm. At frequencies approaching the fundamental diaphragm resonance, and above, this radiation impedance is not negligibly small compared with the acoustic impedance of the microphone, and calculations would have to consider the relation between these impedances for each microphone calibrated. This relation may also be dependent on unknown details (e.g., asymmetry) of the diaphragm motion in ways that are analytically intractable.

However, for frequencies at least two or three octaves below the fundamental resonance of the microphone, theoretical corrections can be expected to provide very good agreement with experimentally determined ones. In particular, Matsui [10,11] has derived theoretical plane-wave free-field corrections at normal incidence for a standard microphone with a recessed diaphragm (which he could also apply to the special case of an unrecessed, or flush-mounted, diaphragm). His analysis assumes that the standard microphone is mounted on a semi-infinite rod that has the same diameter as that of the microphone, and that vibrations of the microphone diaphragm are rotationally symmetric. The Matsui correction applicable to the 1/2-inch MR-112 microphone has been evaluated by Miura et al. [12]. Fortunately, this theoretical correction is at its best at the lowest frequencies of free-field calibration by reciprocity, where technical difficulties posed by the lower signal-to-noise ratios, greater effects of crosstalk, and slight departures from anechoic conditions are greatest, and where the NBS methods for pressure calibration by reciprocity are well-developed and relatively more accurate [13].

Consequently, the adequacy of the uncertainty estimates for the free-field calibration in the most difficult frequency range can be checked by comparing the theoretically and experimentally determined plane-wave free-field corrections for 1/2-inch microphones.

**Figure 22:**
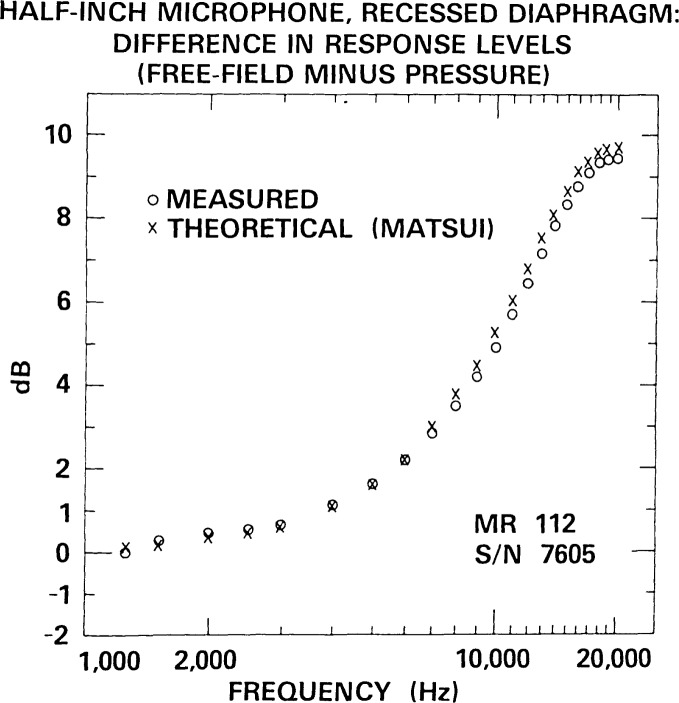
Comparison of experimentally and theoretically determined plane-wave free-field corrections (free-field response level at normal incidence minus pressure response level) for an E. C. L. Type MR-112 1/2-inch microphone with a recessed diaphragm configuration.

Figure 22 shows such a comparison, for an E.C.L. MR-112 1/2-inch microphone with protective grid removed, using the evaluations of Matsui’s theoretical expressions [10,11] by Miura et al. [12] where available, and a low-frequency approximation [11] at other frequencies (e.g., 1.25, 1.5 and 2.5 kHz). The nominal fundmental resonance freqency of the MR-112 is 30 kHz. At frequencies below 7 kHz, e.g., sufficiently below the resonance frequency, the measured and theoretical free-field corrections agree within 0.15 dB (table 6).

**Figure 23:**
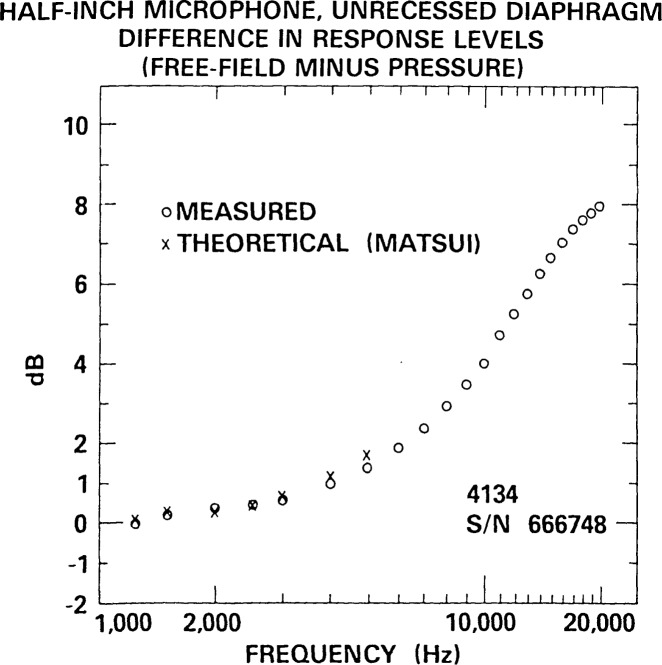
Comparison of experimentally and theoretically determined plane-wave free-field corrections for a Bruel and Kjaer Type 4134 microphone with an unrecessed (flush-mounted) diaphragm configuration.

Figure 23 shows the comparison of free-field plane-wave corrections for a Bruel and Kjaer Type 4134 1/2-inch microphone with protective grid removed. This microphone has a geometry that is approximated by a 1/2-inch diameter cylinder of which the microphone diaphragm constitutes the exposed face, i.e., the diaphragm is unrecessed (flush-mounted). The theoretical plane-wave free-field correction was calculated for this geometry using the approximate low-frequency correction [11] of Matsui. Theoretical corrections were plotted only at frequencies of 5 kHz and lower, because this theoretical approximation is probably losing validity at about 5 kHz, and also because this microphone type has a nominal resonance frequency of 23 kHz, so that experimental and theoretical corrections would not be expected to be nearly equal at frequencies above about 3 to 5 kHz. The agreement (table 6) at frequencies 3 kHz and below is within 0.11 dB, which is excellent. Even at 4 kHz, the agreement is 0.16 dB. Only at 5 kHz, where the agreement is 0.37 dB, and where we have little confidence in the theoretical low-frequency approximation, does divergence become obvious. We rely upon the theoretical diffraction corrections as a check upon experimental results at frequencies below 4 kHz, the most difficult frequency range for free-field calibration by the reciprocity technique. We conclude that at these frequencies the agreement between measured and theoretical plane-wave free-field corrections is consistent with expectations based on the uncertainty estimates for free-field calibration presented herein, the uncertainty (probably at least several hundredths of a decibel) in the theoretical diffraction correction, and the uncertainty in pressure calibration by the reciprocity method in essentially closed couplers (see ref. [13]).

## 8. Considerations Concerning Free-Field Calibrations Traceable to the NBS

For both types of microphone geometry represented in figures 22 and 23, the values of both the theoretical and experimental plane-wave free-field corrections are less than 0.5 dB at frequencies of 2 kHz and below. The smallness of these corrections at 2 kHz and below is a principal reason that the fixed-cost calibration services for free-field calibration of 1/2-inch microphones at NBS begin at a lowest frequency of 2.5 kHz, with lower frequencies available at cost, upon special request.

A number of users of the NBS pressure calibration services send commonly-used types of “1-inch” microphones to the NBS for fixed-cost pressure calibration at frequencies from 50 Hz to 20 kHz, and infer the free-field calibration from this pressure calibration by employing tabulated frequency-dependent values standardized [3,14] for given “1-inch” laboratory standard microphone types and orientations.

However, Koidan and Siegel [15] at the NBS performed measurements of the free-field plane-wave correction for a group of such microphones, and showed that the measured differences in these free-field corrections between individual microphones of the same type were larger than could be attributed to experimental errors. In particular, they found that differences between these corrections measured for each of a group of seven Western Electric Type 640AA and two Electrical Communication Laboratory Type MR 103 condenser microphones were largest at frequencies above about 6 kHz, and that these differences could be as large as 0.9 dB at 10 kHz. Citing a pertinent analysis by Foldy and Primakoff [16], Koidan and Siegel attributed these differences to the dependence of the free-field correction upon the acoustic driving-point impedance and radiation impedance of the microphone for plane waves inci dent on the diaphragm, and noted that different microphones of the same type have somewhat different acoustic impedances. Koidan and Siegel [15] further demonstrated a correlation between their measured free-field corrections and their measured acoustic stiffness constants of individual microphones. These results demonstrate that for measurements of the highest accuracy and precision, the determination of the free-field response level from measurements of the pressure response level and the addition of a “standard” free-field correction should be viewed with caution. For microphones manufactured with close tolerances upon their acoustic impedances, differences in the free-field correction of a given microphone from the standard correction can be expected to be smaller. Koidan and Siegel demonstrated that if data from the two Type 640AA microphones with unusually high diaphragm stiffness and acoustic resistance were excluded from their results, the range of free-field corrections measured at 10 kHz for the remaining group of five Type 640AA microphones and two Electrical Communication Laboratory Type MR 103 microphones was reduced from 0.9 dB to less than 0.4 dB. This expectation is also supported by the mostly unpublished data and analysis that led to the IEC tabulations [14] of such standard corrections. For such microphone types as well as for 1/2-inch microphones, however, more extensive, archivally published data similar to that of Koidan and Siegel [15] are not available, and would be required to establish greater confidence in the uncertainties associated with standardized corrections.

Consequently, for the most demanding applications, we consider that the uncertainties associated with the use of standardized frequency-dependent conversion values at frequencies above a few kHz for specified microphone types and orientations are too large to permit this procedure to be used with the pressure calibration of a given microphone as a substitute for the NBS primary free-field calibration of that microphone.

In particular, we recommend that users of the NBS microphone calibration services who seek the most accurate free-field calibration traceable to the NBS obtain the free-field calibration of a 1/2-inch laboratory condenser microphone by the reciprocity method.

## Figures and Tables

**Table 1:** Grid-to-grid corrections for distance.

Freq.	Grid-to-Grid Correction		Effect of Measured Correction @20 cm	Effect of 2nd Order Fit Correction @20 cm	Effect of Measured Correction @15 cm	Effect of 2nd Order Fit Correction @15 cm
	Measured	2nd Order Fit				

Hz	cm#	cm#	dB*	dB*	dB*	dB*

1001	−0.70	−0.52	0.31	0.23	0.42	0.31
1251	−0.14	−0.51	0.06	0.22	0.08	0.30
1602	−0.52	−0.49	0.23	0.22	0.31	0.29
2003	−0.46	−0.47	0.20	0.21	0.27	0.28
2503	−0.61	−0.45	0.27	0.20	0.36	0.26
3153	−0.52	−0.42	0.23	0.18	0.31	0.25
4005	−0.26	−0.39	0.11	0.17	0.15	0.23
5005	−0.44	−0.35	0.19	0.15	0.26	0.21
6308	−0.34	−0.30	0.15	0.13	0.20	0.18
8009	−0.21	−0.23	0.09	0.10	0.12	0.13
9987	−0.05	−0.17	0.02	0.07	0.03	0.10
12475	−0.14	−0.08	0.06	0.03	0.08	0.05
15968	0.01	0.02	0.00	−0.01	−0.01	−0.01
19973	0.12	0.12	−0.05	−0.05	−0.07	−0.07

# The acoustic center correction for a microphone, referred to the grid, is one-half of the grid-to-grid distance correction. A negative number indicates that the acoustic center is in front of (outside) the exterior surface of the grid.

* The error in a calibration response level produced by the error in level (if uncorrected for acoustic center positions) at the receiving microphone at a given distance and frequency will be one-half of the error in the measured level shown in this table.

**Table 2:** Measured acoustic center positions expressed as grid-to-grid distance corrections, levels measured at specific grid-to-grid distances renormalized (as in figs. 5–18) for effects of these measured acoustic center positions, and worst case grid-to-grid distances.

Freq.	Measured Grid-to-Grid Correction	Level @ 20 cm Renormalized for Measured Grid-to-Grid Correction	Level @ 15 cm Renormalized for Measured Grid-to-Grid Correction	Level @ Worst Case Renormalized for Measured Grid-to-Grid Correction	Worst Case Grid-to-Grid Distance

	cm#	dB+	dB+	dB+	cm*

1001	−0.70	−0.05	0.01	−0.06	23
1251	−0.14	−0.06	−0.09	−0.11	17
1602	−0.52	0.07	0.00	−0.08	25
2003	−0.46	−0.04	0.01	−0.09	22
2503	−0.61	−0.02	0.02	0.06	24
3153	−0.52	−0.06	0.08	−0.09	19
4005	−0.26	−0.04	0.04	0.11	24
5005	−0.44	0.03	0.00	−0.03	24
6308	−0.34	−0.02	−0.01	0.05	22
8009	−0.21	0.02	0.00	0.03	19
9987	−0.05	0.01	0.04	0.04	22
12475	−0.14	−0.01	−0.04	−0.05	19
15968	0.01	0.01	−0.02	0.04	25
19973	0.12	0.00	0.03	0.03	15

Total of absolute values		0.44	0.39	0.87	

Average of absolute values		0.031	0.028	0.062	

* The worst-case spacing was evaluated only for distances between 15 and 25 cm.

# The acoustic center correction for a microphone, referred to the grid, is one-half of the grid-to-grid distance correction. A negative number indicates that the acoustic center is in front of the grid, i.e., outside its exterior surface.

+ The error in a calibration produced by the deviation from zero in level at a given distance and frequency will be one-half of the given deviation.

**Table 3:** Estimated random components of uncertainty two standard deviations from the mean for each term from eqs (3) and (5).

**Term from eqs (3) and (5)**	**Estimated uncertainty (dB)**
[*A*_bd_–*A*_ad_–*A*_ba_]/2	0.03 (5 kHz*⩽f⩽*20 kHz)to 0.09 (1.25 kHz*⩽f⩽* 5 kHz)
10 log_10_ *C*_a_	0.005
10 log*_10_p_s_*,	0.004
10 log_10_ *T*	0.004
20 log_10_*f*	0.0002
4.343 α*r*_ab_	0.007
10 log_10_ *r*_ab_	0.04
polarization voltage (not in eqs (3) and (5))	0.007 dB
alignment (not in eqs (3) and (5))	0.005 dB

**Table 4:** Estimated credible bounds on magnitudes of systematic uncertainties of relevant terms from eqs (3) and (5).

**Term from eqs (3) and (5)**	**Estimated upper bound on uncertainty (dB)**
10_log_ *C*_a_	0.005
10_log_ *r*_ab_	0.015 (5 kHz *⩽f⩽* 20 kHz) to 0.06(1 kHz *⩽f⩽* 5 kHz)

**Table 5:** Overall uncertainty estimates.

Frequency Range (kHz)	Estimated Upper Bound on Magnitude of Systematic Component (dB)	Random (Two Standdard Deviations) (dB)
20 ⩾*f*⩾ 5	0.02	0.05
5 ⩾*f*⩾ 1.25	0.06	0.10

**Table 6:** Differences between measured and theoretical [after Matsui] plane-wave free-field corrections for 1/2-inch microphones with recessed (MR-112) and flush (Bruel & Kjaer 4134) diaphragm configurations (protective grids removed).

Frequency (kHz)	Difference for MR-112 (dB)	Difference for Bruel & Kjaer 4134 (dB)
1.25	−.13	−.11
1.5	.11	.08
2.0	.15	.08
2.5	.09	−.03
3	.04	−.08
4	.05	(−.16)*
5	−.03	(−.37)*
6	−.04	
7	−.10	

* Note: The low-frequency approximation to Matsui’s theoretical correction [10] that was used for the Bruel & Kjaer 4134 loses validity at 4 to 5 kHz. Consequently, the differences at these frequencies show divergence between measured and theoretical values for this microphone.

## Footnotes

1 Bracketed figures refer to literature references.

2 This item is identified only in order to adequately specify the apparatus, and such identification in no way implies that this item is the best or the only commercially available device that could be used for the intended purpose.

## APPENDIX

### Key Instruments* Used in Free-Field Calibration System of Figure 1

Preamplifier: NBS-modified version of Bruel and Kjaer Type 2619. Subsequently to establishment of the system, the Bruel and Kjaer Type 2645 preamplifier with insert-voltage capability has become available commercially. Although not tested in our system, this preamplifier could reasonably be expected to provide comparable performance to the NBS-modified 2619, provided that the manufacturer’s recommendation for compatibility of microphones (especially with regard to resistance of the microphone insulator) used with the 2645 are followed.

Measuring amplifier: Briiel and Kjaer Type 2608, modified for precise control of polarization voltage by means of a 10-turn precision potentiometer.

One-third octave band-pass filter: Bruel and Kjaer Type 5004.
Lock-in amplifier: Princeton Applied Research Model 129A.
Digital voltmeter (reading dc output of lock-in amplifier): Keithley Model 172.
Digital voltmeter (used to check dc polarization voltages): Keithley Model 619 Electrometer/Multimeter.

Oscillator: Krohn-Hite Model 4025R (used mostly in earlier stages of work) *or* (more recently) Hewlett-Packard Model 3325A Synthesizer/Function Generator with high stability frequency reference option. The output of the 3325A is suitably attenuated (not shown in fig. 1) before entering the isolation transformers and tuning input of the lock-in amplifier.

Amplifier (at output of Oscillator): Hewlett-Packard Model 467A.
Precision Attenuator: Daven Spec. 8304.
Barometer: Wallace & Tiernan Model FA 139210.
Isolation transformers: Gertsch Model ST-200.
